# High concentrations of dried sorghum stalks as a biomass feedstock for single cell oil production by *Rhodosporidium toruloides*

**DOI:** 10.1186/s13068-014-0190-y

**Published:** 2015-01-22

**Authors:** Leonidas Matsakas, Nemailla Bonturi, Everson Alves Miranda, Ulrika Rova, Paul Christakopoulos

**Affiliations:** Biochemical Process Engineering, Division of Chemical Engineering, Department of Civil, Environmental and Natural Resources Engineering, Luleå University of Technology, SE-971 87 Luleå, Sweden; Department of Materials and Bioprocess Engineering, School of Chemical Engineering, State University of Campinas, Av. Albert Einstein, 500, 13083-852 Campinas, SP Brazil

**Keywords:** Single cell oil, *Rhodosporidium toruloides*, Sweet sorghum, Enzymatic saccharification, Biodiesel

## Abstract

**Background:**

Environmental crisis and concerns for energy security have made the research for renewable fuels that will substitute the usage of fossil fuels an important priority. Biodiesel is a potential substitute for petroleum, but its feasibility is hindered by the utilization of edible vegetable oil as raw material, which is responsible for a large fraction of the production cost and fosters the food versus fuel competition. Microbial oils are an interesting alternative as they do not compete with food production, and low cost renewable materials could serve as raw materials during cultivation of microorganisms. Sweet sorghum is an excellent candidate as substrate for microbial oil production, as it possesses high photosynthetic activity yielding high amounts of soluble and insoluble carbohydrates, and does not require high fertilization and irrigation rates.

**Results:**

Initially the ability of sweet sorghum to fully support yeast growth, both as a carbon and nitrogen source was evaluated. It was found that addition of an external nitrogen source had a negative impact on single cell oil (SCO) production yields, which has a positive effect on the process economics. Subsequently the effect of the presence of a distinct saccharification step on SCO was examined. The presence of an enzymatic saccharification step prior to SCO production improved the production of SCO, especially in high solid concentrations. Removal of solids was also investigated and its positive effect on SCO production was also demonstrated. When juice from 20% w/w enzymatically liquefied sweet sorghum was used as the raw material, SCO production was 13.77 g/L. To the best of our knowledge this is one of the highest SCO titers reported in the literature when renewable raw materials were utilized.

**Conclusions:**

The use of sweet sorghum at high solid concentrations as a feedstock for the efficient production of SCO by Rhodosporidium toruloides was demonstrated. Moreover, addition of enzymes not only led to liquefaction of sweet sorghum and permitted liquid fermentation, but also enhanced lipid production by 85.1% and 15.9% when dried stalks or stalk juice was used, respectively.

## Background

Current industrialization, environmental problems, and the rate of depletion of fossil fuels have raised the global demand for alternative and renewable sources of energy [1,2]. Unlike electricity production, which has several renewable options available, the transportation sector is usually limited to diesel and gasoline, with ethanol and biodiesel being the two most promising renewable fuels to replace them.

Biodiesel is a renewable, non-toxic, and biodegradable fuel [3]. It is produced by the transesterification of triacylglycerols (TAGs) from vegetable oils and animal fats into fatty acid methyl or ethyl esters (FAMEs), for which edible plant oils (sunflower, rapeseed, and soybean) have been the main raw material. Recently, it has become evident that biofuel production should be based on non-food crops, agro-industrial wastes, and renewable resources to avoid direct competition with food production [4]. In fact, these concerns have turned the efforts of researchers to finding alternative sources of TAGs, such as microbial oils, so called single cell oils (SCOs), for the production of biodiesel. In order to be classified as oleaginous, the microorganism should have the ability to accumulate at least 20% of the total dry cell mass as lipids [5], and can be a bacterium, yeast, filamentous fungus, or algae [6].

Yeasts have advantages over other microorganisms due to the higher availability of oleaginous candidates compared to bacteria, the shorter cultivation time compared to other fungi, and the ease of scale-up compared to algae [7]. The yeast *Rhodosporidium toruloides* is an excellent candidate for SCO production, as it can accumulate high quantities of lipids when grown on different substrates such as pure sugars [8,9], crude glycerol [10], cassava starch [11], or Jerusalem artichoke [12]. Furthermore, this yeast is capable of producing high-value products such as carotenoids [13].

Techno-economic evaluations of the biodiesel production using the oil from *R. toruloides* showed that the cost of glucose and yeast extract respectively represent 80% and 16% of the raw material costs [4]. For the technology of biodiesel production from microbial oil to become economically feasible, it is crucial to find a low cost and widely available substrate that can provide sugars and nitrogen for microbial growth and accumulation of oil.

Sweet sorghum (*Sorghum bicolor* (L.) Moench) is a C4 plant with several interesting features, such as high biomass yield per hectare, increased drought resistance, low fertilization requirement, high adaptability to different climates and soils, and short growth period (between three and five months). Sweet sorghum should therefore be a suitable feedstock for innumerable bioprocesses, due to the high yields of lignocellulosic biomass and fermentable sugars [14]. Its potential as a feedstock has already been shown for ethanol production [15,16] and methane production [17]. There are some studies concerning the use of sweet sorghum for lipids production as whole stalks [18], juice [19,20], and the lignocellulosic fraction after sugars removal, the so-called bagasse [21,22]. The main obstacle when sweet sorghum is used is its low stability during storage due to the high concentration of soluble sugars present. Soluble sugars can be protected from microbial degradation if the stalks are dried, which has already been shown [16,23]. The aim of this work was therefore to evaluate for the first time the production of lipids by *R. toruloides*, using dried sweet sorghum stalks as feedstock.

## Results and discussion

### Effect of nitrogen addition on single cell oil production

Sweet sorghum stalks contain high amounts of soluble carbohydrates (glucose, fructose, and sucrose) and insoluble carbohydrates (cellulose and hemicellulose), which could be converted to monomeric sugars and used by the yeast. Proteins are also present in the stalks and could be partly used by the yeast as a nitrogen source, resulting in an initial carbon-to-nitrogen (C:N) ratio of approximately 60:65 [18]. This value might be a challenge during SCO production, as higher C:N ratios are generally required in order to obtain high yields of SCOs. On the other hand, not all proteins are readily available to different fermenting microorganisms, which could in turn result in a higher actual C:N ratio.

In order to evaluate the ability of *R. toruloides* to use sweet sorghum proteins as a nitrogen source, the effect of the addition of external nitrogen (yeast extract), at a concentration equivalent to 0.3 g per 100 g of stalks, was evaluated at an initial sweet sorghum concentration of 8.7% w/w. Yeast extract was chosen as the nitrogen source as it has previously been shown that organic nitrogen is more favorable than inorganic nitrogen for accumulation of SCO by *R. toruloides* [24,25]. Addition of even this low amount of external nitrogen resulted in a decrease in SCO production from 6.12 g/L to 2.60 g/L. Thus, it can be concluded that *R. toruloides* is capable of efficiently using the proteins present in the stalks, which in turn has a positive effect on the economics of the process, as the addition of nitrogen significantly increases the cost of SCO production.

### Effect of the initial sweet sorghum concentration, in the presence or absence of a distinct saccharification step, on single cell oil production yields

**Figure 1:**
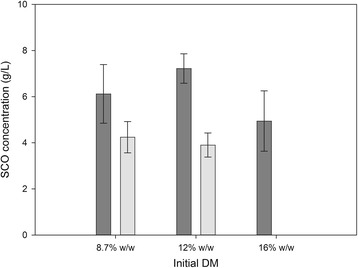
**Effect of sweet sorghum concentration in the presence or absence of enzymatic saccharification on single cell oil (SCO) production.** Effect of initial sweet sorghum concentration at a range from 8.7 to 16% w/w in the presence (dark grey) or absence (light grey) of enzymatic saccharification on the production of SCO. DM, dry matter.

**Figure 2:**
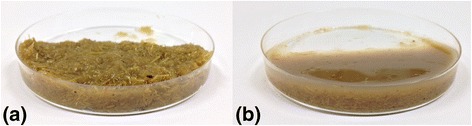
**Effect of enzymatic saccharification on sweet sorghum at 16% w/w. (a)** Without the application of enzymatic saccharification and **(b)** after enzymatic saccharification.

The effect of the initial sweet sorghum content on the production of SCO was tested in the 8.7 to 16% w/w range. The maximum lipid production was obtained at 12% w/w (Figure 1). A further increase in the solids content resulted in inhibition of growth, probably due to inefficient air-transfer properties of the high solids mash. In contrast, when a distinct enzymatic saccharification step was used, this resulted in efficient growth of yeast even at 16% w/w sweet sorghum content, and higher SCO production yields for all solids contents, underscoring the great importance of saccharification. As can be seen in Figure 2, at the highest solids content of 16% w/w, enzymatic saccharification resulted in liquefaction of the material and, consequently, better mixing of the yeast. The viscosity of lignocellulosic substrates is known to decrease as a result of cellulolytic activity, with the most probable reason being the collapse of structure and subsequent loss of water-binding capacity upon the degradation of cellulose [26]. Moreover, enzymatic saccharification facilitates the release of soluble sugars from cellulose and hemicellulose, and consequently improves the C:N ratio.

### Effect of removal of solids on higher single cell oil production

In a previous report on SCO production by the yeast *Lipomyces starkeyi*, it was found that the yeast was unable to grow in the presence of the solids from sweet sorghum stalks, even at a low solids content. The removal of solids resulted in efficient growth and lipid production [27], the most probable reason being that the presence of solids reduced the oxygen transfer efficiency.

**Figure 3:**
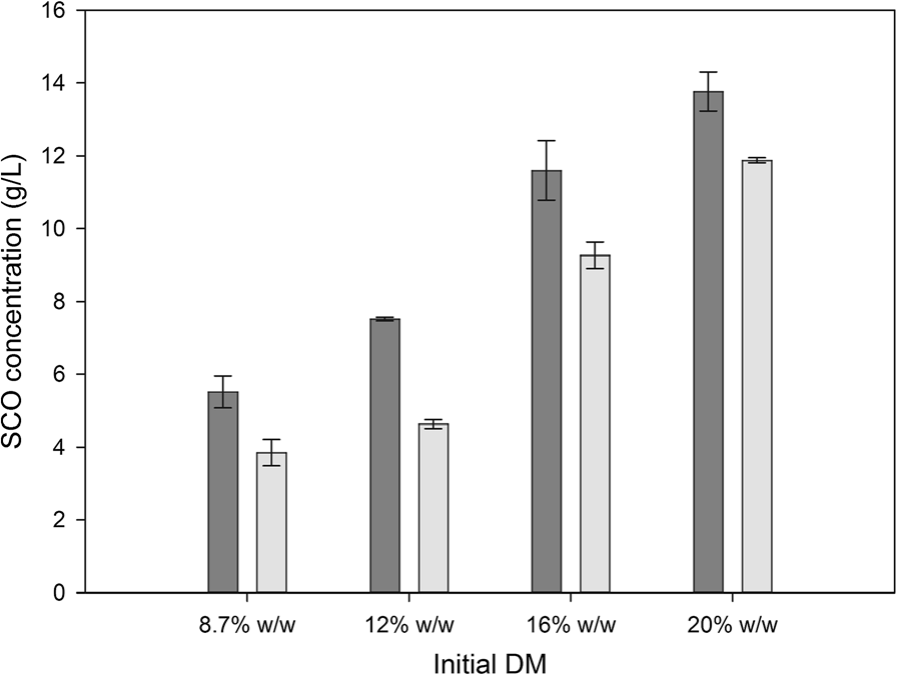
**Effect of solids removal on single cell oil (SCO) production in the presence or absence of enzymatic saccharification.** Effect of sweet sorghum concentration at a range between 8.7 and 20% w/w on SCO production in the presence (dark grey) or absence (light grey) of enzymatic saccharification. Solids were removed prior to cultivation.

**Figure 4:**
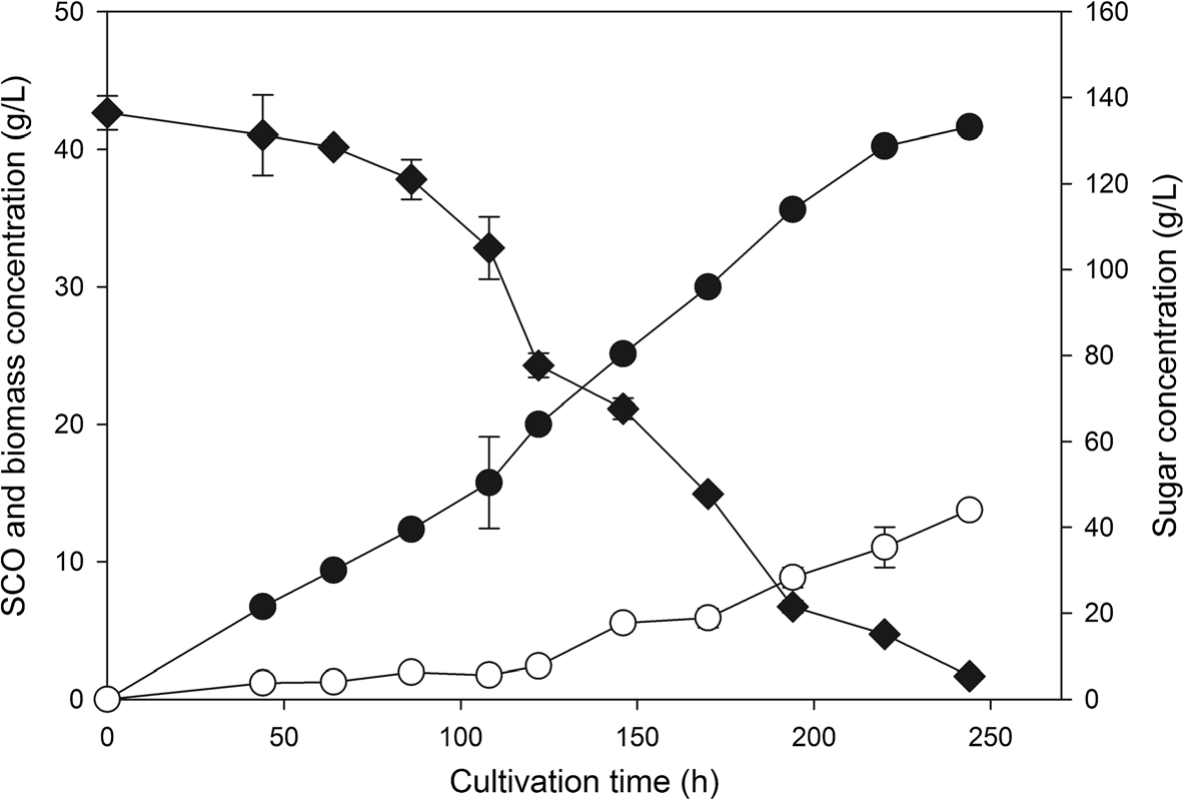
**Time course of single cell oil (SCO) production on juice obtained from 20% w/w solids concentration.** Time course of sugars consumption (diamond), biomass (filled circle) and lipid (circle) concentration when *R. toruloides* was cultivated on sweet sorghum juice that came from 20% w/w sweet sorghum concentration.

For this reason, we also evaluated the effect of the removal of solids on SCO production in this study, both with and without a distinct saccharification step. We found that the removal of solids after the saccharification step resulted in enhanced SCO production even at high concentrations of sweet sorghum, such as 20% w/w (Figure 3). The cell growth, sugar consumption, and lipid production at a solids concentration of 20% are shown as a function of time in Figure 4. It is worth mentioning that glucose and fructose co-utilized almost at the same rate, whereas sucrose was totally hydrolyzed by the invertase activity exhibited by Novozym^®^188 to glucose and fructose (data not shown). The maximum lipid concentration (13.77 g/L) with lipid content of 33.1% w/w was observed on day 10. This production value is higher than most of the SCO concentrations reported in the literature when renewable resources have been used as raw materials (Table 1). Moreover, in this work an enzymatic treatment of an energy crop was used, which offers advantages over the acid hydrolysis of lignocellulosic materials such as low energy consumption due to the mild process requirements, high sugar yields, no requirement for detoxification, and no unwanted wastes. The SCO yield was equal to 0.105 g/g of consumed sugars, whereas the biomass formation yield was 0.318 g/g, and the lipid productivity reached 1.377 g/L per day. The use of an enzymatic saccharification step resulted in an increase in SCO production of 15.9% relative to the experiment without enzymatic saccharification.

**Table 1:** **Comparison of single cell oil production from renewable raw materials**

**Microorganism**	**Raw material**	**Lipid concentration (g/L)**	**Reference**
*Rhodosporidium toruloides*	Detoxified dilute sulfuric acid pretreated wheat straw	2.4	[28]
*Yarrowia lipolytica*	Detoxified hydrochloric acid pretreated sugarcane bagasse	6.7	[29]
*Rhodotorula glutinis*	Sulfuric acid hydrolyzed tree leaves	4.7	[30]
*Trichosporon fermentans*	Detoxified dilute sulfuric acid pretreated rice straw	11.5	[31]
*Rhodotorula glutinis*	Monosodium glutamate wastewater	5.0	[32]
*Chlorella protothecoides*	Sweet sorghum juice	2.9	[19]
*Schizochytrium limacinum*	Sweet sorghum juice	6.9	[20]
*Trichosporon fermentans*	Detoxified sulfuric acid treated sugarcane bagasse hydrolysate	15.8	[33]
*Cryptococcus curvatus*	Lime pretreated sweet sorghum bagasse	2.6	[22]
*Cryptococcus curvatus*	Dilute sulfuric acid pretreated sweet sorghum bagasse	4.3	[34]
*Rhodosporidium toruloides*	Juice from enzymatically saccharified sweet sorghum	13.8	This work

Table 2 shows the fatty acid profile of the SCO produced by *R. toruloides* when cultured on juice obtained from 20% w/w sweet sorghum solution. The predominant fatty acid is oleic acid, followed by palmitic acid. A high concentration of oleic acid is advantageous, as it is considered to be beneficial for the production of biodiesel [35].

**Table 2:** **Fatty acids composition**

**Fatty acid**	**% concentration (w/w)**
C14:0	0.71
C16:0	29.18
C18:0	6.56
C18:1 (n-9)	55.78
C18:2 cis12	7.68

The fatty acid composition of the obtained single cell oil during cultivation of *R. toruloides* on juice from 20% w/w liquefied sweet sorghum stalks.

## Conclusions

We have demonstrated a unique possibility of using sweet sorghum stalks at high dry matter content in order to produce high SCO yields. Sweet sorghum stalks were capable of fully supporting yeast growth without any need for an external nitrogen source. Incorporation of a distinct enzymatic saccharification step not only led to liquefaction of sweet sorghum and permitted submerged cultivation at high solids content, but also had a positive effect on the SCO yields. Cultivation of the yeast was able to be performed in the presence of solids, also resulting at high SCO concentration. On the other hand, removal of solids significantly enhanced the production of SCO, which reached a maximum concentration of 13.77 g/L.

## Methods

### Raw material, microorganism, and enzyme solutions

Sweet sorghum (*Sorghum bicolor* (L.) Moench) belonging to the Keller cultivar was kindly donated by Professor George Skarakis (Department of Crop Science, Agricultural University of Athens). Seeds and leafs were removed by hand and the stalks were preserved at −20°C until used. Dried sweet sorghum stalks were prepared as previously described [16] and milled at 0.75 mm particles. The composition of the dried stalks in carbohydrates was (% w/w): glucose, 8.2; fructose, 8.1; sucrose, 34.4; cellulose, 19.6; and hemicellulose, 15.2 [16].

*R. toruloides* CCT 0783 was obtained from Coleção de Culturas Tropicais (Campinas, Brazil). For the saccharification of sweet sorghum, a mixture of Celluclast® 1.5 L (cellulases from *Trichoderma reesei*) and Novozym® 188 (β-glucosidase from *Aspergillus niger*) from Novozymes A/S (Bagsværd, Denmark) at a 5:1 v/v ratio was employed. The activity of the mixture was measured according to standard assay [36] and found to be 83 filter paper unit (FPU)/mL.

### Yeast maintenance and inoculum preparation

Yeast was maintained in agar plates containing medium of the following composition: glucose, 20 g/L; meat peptone, 10 g/L; yeast extract, 10 g/L; KH_2_PO_4_, 6 g/L; Na_2_HPO_4_, 2 g/L; and agar, 20 g/L, and maintained at pH 5.5. Prior to any experiment one loop of the yeast was inoculated in 250 mL Erlenmeyer flasks containing 50 mL of pre-culture medium (which was the same composition as in the agar plates, except for the agar) and incubated at 30°C and 200 rpm for 24 hours.

### Preparation of sweet sorghum stalks and single cell oil production

Sweet sorghum stalks were saccharified at 50°C at the optimized conditions which were previously described [16]. During the experiments where the solids were removed, sweet sorghum was squeezed through a coating sheet at the end of the saccharification, followed by centrifugation. The obtained liquids were sterilized and utilized for the cultivation of the yeast. When no saccharification was applied, sweet sorghum stalks were presoaked at 50°C for two hours in order to facilitate sugars extraction, prior to solids removal. When saccharification was included, Celluclast® 1.5 L was added at the start-up of the saccharification, whereas Novozym®188 was added at the start-up of the cultivation. The addition of Novozym®188 at the start-up of the cultivation and not during the hydrolysis was done because it was previously found that it contains invertase activity [16]. The presence of invertase would result in sucrose hydrolysis and, in turn, in increased inhibition of the enzymes by glucose.

Cultivation broth was supplemented with 1.5 g/L of MgSO_4_.7H_2_O and KH_2_PO_4_ each, as well as with 1% of trace elements solution which was prepared as elsewhere described [37] and incubated at 30°C and 200 rpm after inoculation with 5% v/v of the pre-culture media. At different time intervals samples were taken and analyzed for total sugars, biomass and SCO. All experiments were done in duplicates.

### Analytical methods

Prior analysis samples were centrifuged in order to remove yeast cells and sweet sorghum particles (if present). Supernatant was used for total sugars quantification by the 3,5-dinitrosalicylic acid (DNS) method [38]. In order to enable sucrose determination by DNS, sucrose was hydrolyzed under acidic conditions for 15 minutes at 70°C. The solids were washed with distilled water in order to remove remaining sugars and salts and centrifuged again. During the experiments with solids removal, the yeast biomass was determined after drying at 80-90°C until constant weight. For the experiments with the presence of solids the yeast biomass was determined by plating samples on agar plates and incubating them at 30°C. The number of viable cells was expressed as colony forming units per mL (cfu/mL) and was correlated to cell dry mass (g/L) using a calibration curve. SCO was extracted from the dried samples by a mixture of chloroform:methanol at a 2:1 v/v ratio [39], and quantified after solvent evaporation under vacuum using a rotary evaporator. It is worth mentioning that when solids were present, the concentration of lipids presented in the stalks was removed from the following samples, in order to present the net production by the yeast only.

Analysis of fatty acid composition was performed in a gas chromatography apparatus (Varian CP-3800, coupled to a capillary column WCOT (Wall coated open tubular) fused silica 100 m × 0.25 mm coating CPSIL 88 for FAME, Agilent Technologies, Santa Clara, CA, USA,), after forming FAMEs from the yeast oil according to the method described by Appelqvist [40]. The temperatures of detector and injector were 270°C and 300°C, respectively. The column oven temperature was initially kept at 175°C for 26 minutes and subsequently increased to 205°C at a rate of 2°C/min, and remained at this temperature for 24 minutes. The carrier gas was helium, at a flow rate of 30 mL/min.

## Abbreviations

C:N: Carbon to nitrogen
cfu: Colony forming units
DM: Dry matter
DNS: 3,5-dinitrosalicylic acid
FAME: Fatty acid methyl ester
FPU: Filter paper unit
SCO: Single cell oil
TAG: Triacylglyceride
